# Effect of pioglitazone versus insulin glargine on cardiac size, function, and measures of fluid retention in patients with type 2 diabetes

**DOI:** 10.1186/1475-2840-8-15

**Published:** 2009-03-20

**Authors:** Mozhgan Dorkhan, Magnus Dencker, Martin Stagmo, Leif Groop

**Affiliations:** 1 Department of Clinical Sciences, Division of Diabetes & Endocrinology, Lund University, Malmö University Hospital, Malmö, Sweden; 2 Department of Clinical Sciences, Unit of Clinical Physiology and Nuclear Medicine, Lund University, Malmö University Hospital, Malmö, Malmö, Sweden; 3 Department of Clinical Sciences, Division of Cardiology, Lund University, Malmö University Hospital, Malmö, Sweden

## Abstract

**Background:**

Both insulin and thiazolidinediones (TZDs) are effective in the treatment of hyperglycaemia and amelioration of insulin resistance in type 2 diabetes but have side effects including weight gain and fluid retention. The use of TZDs has been further hampered by the risk of adverse cardiovascular events including heart failure. The present study evaluated the effect of pioglitazone or insulin glargine on cardiac function and size as well as on surrogate markers of fluid retention such as weight, haemoglobin and natriuretic peptides.

**Methods:**

Thirty patients with inadequate glycaemic control on metformin and sulfonylurea were randomised to receive add-on therapy with insulin glargine or pioglitazone for 26 weeks. Echocardiographic data and blood samples were collected from the two groups before the start of the treatment and after 26 weeks. Left ventricular end-diastolic and left atrial end-systolic volumes were quantified, weight measured and blood samples analyzed.

**Results:**

After 26 weeks of treatment, the changes in HbA1c, weight and haemoglobin were similar between the two groups. HDL increased significantly in the pioglitazone group. While there was an increase in natriuretic peptides in the pioglitazone group (NT-proBNP 11.4 ± 19.6 to 22.8 ± 44.0, p = 0.046), the difference between the treatment groups was not significant. Left ventricular end-diastolic volume increased by 11% and left atrial end-systolic volume by 17% in the pioglitazone group (Both, p < 0.05, between treatment groups). There was a borderline significant increase in ejection fraction in the pioglitazone group.

**Conclusion:**

This randomised pilot-study showed that six-month treatment with pioglitazone induced significant increases in natriuretic peptides and alterations of cardiac size. These changes were not observed with insulin glargine, which also is known to induce fluid retention. Larger randomised trials are warranted to confirm these findings.

## Introduction

The hyperglycaemia in type 2 diabetes (T2D) is associated with enhanced risks of micro- and macrovascular complications. The continuous deterioration of metabolic control is considered independent of mode of therapy and ascribed to deterioration of both insulin secretion and action. Consequently, there is no widely agreed consensus on how to intensify therapy in patients who fail on initial treatment with metformin and/or a sulfonylurea, especially whether to add a third oral agent, e.g., thiazolidinedione (TZD) or insulin. Both treatment alternatives are considered to result in weight gain and fluid retention. While insulin therapy requires injections and can be associated with hypoglycaemia, TZD therapy is associated with increased risk of heart failure. In a recent meta/teleo-analysis Singh et al. concluded that heart failure might occur at both high and low doses, usually weeks to months after initiating TZDs and in patients without a history of heart failure [1]. There are, to our knowledge, no studies that have investigated whether there are any differences between these two treatment alternatives on cardiac size and function in patients with T2D. The present study thus evaluated whether pioglitazone or insulin glargine added to metformin and sulfonylurea differ in their effects on cardiac size and function as well as in haematological signs of fluid retention and cardiac dysfunction.

## Patients and methods

Thirty patients with T2D and inadequate glycaemic control were included in a 26-week randomised open-label trial. Inadequate glycaemic control was defined as treatment with metformin and sulfonylurea/meglitinide in doses > 50% of maximum recommended doses and HbA1c > 6.2% measured with Mono-S method (= 7% National Glycohemoglobin Standardisation Program, NGSP). Patients with known heart failure or clinical signs of heart failure (New York Heart Association class II–IV) were excluded. Also excluded from the study were patients with significant valvular dysfunction (defined as more than mild regurgitation or presence of valvular stenosis), reduced ejection fraction EF (< 50%) or inappropriate acoustic window. Patients who met the criteria for the study were randomly assigned to receive add-on therapy with insulin glargine or pioglitazone for 26 weeks. Insulin was up-titrated to achieve fasting plasma glucose < 6 mmol/l. Pioglitazone was increased to 45 mg/day after 16 weeks if HbA1c > 6.2%. Weight and height were measured, and BMI was calculated at inclusion and after 26 weeks of treatment. Blood samples were collected for measurement of haemoglobin, liver enzymes, plasma lipids, and natriuretic peptides (BNP and NT-proBNP). Blood samples were drawn after an overnight fast, and after at least 10 minutes rest in the supine position.

### Echocardiography

Standard transthoracic echocardiography examinations were performed with Sonos 5500 (Philips, Andover, MA, USA) at baseline and after 26 weeks of treatment. A single observer blinded to all clinical data performed the echocardiography measurements, which were performed twice with the mean value used in all analysis. From 2-D echocardiography, left ventricular end-diastolic diameter (LVDD), posterior wall thickness (POST) and interventricularseptum thickness (IVS) were measured and left ventricular mass (LVM) was calculated from the formula LVM = 0.8 × 1.04 × (LVDD+IVS+POST)^3^-LVDD^3 ^+0.6 [2]. Left ventricular end-diastolic volume (LVD vol), left ventricular end-systolic volume (LVS vol), left atrial end-systolic volume (LA vol), and EF were quantified by biplane method of discs [2]. Percent change from baseline measurements were calculated.

### Assays

Plasma glucose was measured with a HemoCue glucose analyser (Hemocue, Ängelholm, Sweden). HbA1c was analyzed using the Variant II chromatographic method from Bio-Rad (ref. 4–5.3%) measured by Swedish Mono-S (high performance ion-exchange liquid chromatography). BNP was measured with an immunoassay system from Beckman (Biosite, Ca, USA) with a CV of 3.5% and NT-proBNP using an immunometric method (Hitachi Modular-E, Roche) with a CV of 4.4%.

### Statistical methods

Data are presented as means ± SD where appropriate. Non-parametric tests were used to test differences in changes of measured variables within and between treatment groups. A two-side significance level of < 0.05 was considered statistically significant. Statistical analyses were carried out using SPSS version 15 and Microsoft office Excel 2003.

## Results

All 30 patients completed the study. There were no significant differences in patients' anthropometrical characteristics at baseline or at the end of the study (Table 1). After 26 weeks of treatment, the reduction in HbA1c was similar in groups as was weight gain and reduction in haemoglobin levels. Pioglitazone, but not insulin glargine resulted in an increase in HDL concentrations (1.10 ± 0.25 to 1.3 ± 0.31, p < 0.01 vs. 1.1 ± 0.37 to 1.1 ± 0.36, p = ns, p between groups = 0.013).

**Table 1:** Subjects' anthropometric and laboratory characteristics at baseline and after 26 weeks of treatment.

	**Pioglitazone**			**Insulin glargine**			**Δ pioglitazone vs. Δ insulin glargine** **P**
	**Week 0**	**Week 26**	**P**	**Week 0**	**Week 26**	**P**	
	15			15			
Men/women	11/4			9/6			
Age(years)	60.8 ± 7.1			61.5 ± 8.2			
Diabetes Duration (years)	11.1 ± 6			9.5 ± 7.5			
BMI (kg/m^2^)	30.8 ± 5.5	31.9 ± 5.9	0.001	31.6 ± 6.1	32.5 ± 6.2	0.01	0.37
HbA_1c _(%)	8.1 ± 1.5	6.8 ± 1.2	0.001	8.3 ± 1.4	6.1 ± 0.7	0.001	0.23
Haemoglobin (g/l)	137.5 ± 11.4	127 ± 12.0	0.001	137.1 ± 10.2	132 ± 12.0	0.02	0.13
Cholesterol (mmol/l)	4.4 ± 0.9	4.2 ± 0.7	0.17	4.3 ± 1.0	4.1 ± 1.0	0.32	0.65
HDL (mmol/l)	1.1 ± 0.2	1.3 ± 0.3	0.01	1.1 ± 0.4	1.1 ± 0.4	0.49	0.01
LDL (mmol/l)	2.4 ± 0.9	2.3 ± 0.5	0.64	2.3 ± 0.7	2.4 ± 0.7	0.46	0.48
TG (mmol/l)	1.8 ± 0.8	1.5 ± 1.0	0.002	1.9 ± 1.3	1.5 ± 1.0	0.009	0.43
ALT (μcat/l)	0.7 ± 0.7	0.4 ± 0.3	0.04	0.5 ± 0.2	0.5 ± 0.2	0.12	0.87
Systolic blood pressure	142 ± 16	134 ± 10	0.04	146 ± 17	132 ± 13	0.02	0.39
Diastolic blood pressure	84 ± 8	77 ± 7	0.02	81 ± 8	73 ± 8	0.007	0.57
BNP (pmol/l)	6.9 ± 5.2	14.1 ± 16.7	0.10	9.7 ± 12.9	9.7 ± 11.6	0.41	0.51
NT-proBNP (ng/l)	11.4 ± 19.6	22.8 ± 44.0	0.046	13.6 ± 20.0	10.1 ± 9.5	0.97	0.23

Values are mean ± SD. There were no significant differences between subjects' characteristics at start or after 26 weeks of treatment.

Baseline BNP and NT-proBNP correlated with change during intervention in all subjects (r = 0.79, p < 0.01). There was a doubling of BNP and NT-proBNP concentrations in the pioglitazone group (6.9 ± 5.2 to 14.1 ± 16.7, p = 0.10 and 11.4 ± 19.6 to 22.8 ± 44.0, p = 0.046) and no change in the glargine group (9.7 ± 12.9 to 9.7 ± 11.6, p = 0.41 and 13.6 ± 20.0 to 10.1 ± 9.5, p = 0.97). The difference between the treatment groups, however, was not significant. The increase in BNP and NT-proBNP correlated inversely with the changes in haemoglobin (r = -0.38, p = 0.041 and r = -0.47, p = 0.01) in the whole group. The inverse correlation between increase in NT-proBNP and the change in haemoglobin was even greater in the group treated with pioglitazone (r = -0.59, p = 0.02) while there was no correlation in the group treated with insulin glargine (r = -0.016, p = 0.95). The NT-proBNP values at baseline correlated strongly with changes from baseline during treatment with pioglitazone (r = 0.63, p < 0.012). None of the subjects developed clinical heart failure. There were no significant differences in baseline echocardiography measurements between the groups, whereas significant increase in LVD vol and LA vol could be observed after six-month treatment with pioglitazone. There were no differences in change of LVM or EF between the two groups. Table 2 displays baseline characteristics ( ± SD) and percent change in echocardiography data after 26-week treatment. There were significant correlations between changes in BNP and NT-proBNP versus change in LA vol in the whole group (r = 0.42 and r = 0.56, p < 0.05). There were no significant correlations between changes in BNP and NT-proBNP versus change in LVD vol in the whole group (r = 0.08 and r = 0.16, both NS).

**Table 2:** Subjects' echocardiography data at baseline and after 26 weeks of treatment.

Value	Pioglitazone group (n = 15)	Insulin glargine group (n = 15)	P-value
LVDD (mm)	47.6 ± 4.0	48.7 ± 4.5	0.37
IVS (mm)	10.2 ± 0.9	10.4 ± 1.6	0.85
POST (mm)	9.0 ± 1.0	9.1 ± 1.4	0.95
LVM (g)	160 ± 33	173 ± 56	0.55
LVD vol (ml)	104 ± 23	101 ± 21	0.88
LVS vol (ml)	39 ± 11	36 ± 11	0.34
EF (%)	62 ± 5	65 ± 5	0.21
LA vol (ml)	55 ± 14	58 ± 19	0.56
Change LVM (%)	7 ± 14	1 ± 11	0.25
Change LVD vol (%)	11 ± 19	1 ± 5	**0.02**
Change LVS vol (%)	2 ± 15	2 ± 13	0.74
Change EF (%)	6 ± 10	0 ± 6	0.09
Change LA vol (%)	17 ± 17	2 ± 6	**<0.01**

Although one patient in the pioglitazone group developed a significant mitral regurgitation during treatment, the condition was reversible, shown by the results of an echocardiography examination six months after discontinuation of pioglitazone medication [3].

## Discussion

Both pioglitazone and insulin glargine added to treatment with metformin and sulfonylurea/meglitinide in patients with T2D was well tolerated and improved glycaemic control. In both groups a similar weight gain was observed. In addition, pioglitazone therapy led to alterations in cardiac volumes. The pioglitazone therapy resulted in a significant increase in LVD vol and LA vol, as measured by echocardiography as well as increase in levels of BNP and NT-proBNP. However, the difference in changes of natriuretic peptides between groups was not significant, probably due to the small sample size and the high intra-individual variation. Pioglitazone also increased HDL cholesterol in keeping with previous studies. To our knowledge, this is the first study in patients with T2D demonstrating differences in the effect of insulin vs. a TZD on cardiac function. The findings should, however, be viewed with caution due to the modest sample size.

BNP and NT-proBNP are peptide hormones released from the cardiac ventricles in response to myocyte stretch, and have generated attention in recent years as potential diagnostic and prognostic markers for cardiac disease. Natriuretic peptides have been shown to correlate with cardiac dysfunction, and NT-proBNP has been shown to be independent risk marker for cardiovascular disease in patients with diabetes [4,5]. For example, a reduction of 10 pg/ml in Nt-proBNP is associated with a 1% relative reduction in the cardiovascular endpoints in patients with type 1 diabetes and microalbuminuria [4]. There is consensus that natriuretic peptides are useful in screening for cardiovascular disease, especially in symptomatic patient populations [6]. Furthermore, natriuretic peptides carry powerful prognostic information [6]. There are, however, currently no clear algorithms on how these biomarkers should be implemented into clinical practice in patients with T2D when choosing add-on treatment or monitoring the effect of the glucose-lowering agents.

There were no significant differences for echocardiography measurements at baseline between the two groups, whereas significant increase in LVD vol and LA vol could be observed after 26-weeks treatment with pioglitazone. These changes were of moderate magnitude as LVD vol increased by 11% and LA vol by 17% in the pioglitazone group. There where no significant differences in EF at baseline and only a borderline significant increase in the pioglitazone group after 26-weeks treatment. There are, to our knowledge, no previous randomised studies that have evaluated the effect of pioglitazone versus insulin glargine on cardiac function and size in patients with T2D. St John Sutton et al. evaluated the effect of rosiglitazone versus glyburide in 203 patients with T2D [7]. In this study, a similar increase in LVD vol could be detected in both groups (12% and 19%, respectively), whereas no change in EF could be detected. The borderline significant increase in EF observed in the pioglitazone group in our study could be due to volume overload, but these findings should be interpreted with caution. The findings of increased LVD vol by St John Sutton et al. are in line with the findings of the present investigation that also found an increase in LA vol. The latter finding is of interest since LA vol has been found to be a potent prognosticator in a variety of cardiac disorders as well as in the general population [8]. Moreover, the finding that changes in BNP and NT-proBNP correlated with change in LA vol is of interest and, in part, support the use of BNP or NT-proBNP as markers to monitor cardiac alterations in this patient population. The effect of pioglitazone was investigated in a small T2D population by Hirayama and co-workers [9]. This investigation included 10 male T2D hypertensive and 12 normotensive patients, from which echocardiographic data was gathered at baseline. After a 6-month treatment with pioglitazone, fractional shortening (a simple way of measuring EF) did not change. There was, however, a significant decrease in LVM in 12 normotensive patients. The effect on LVD vol or LA vol was not investigated. There are still other studies that have investigated possible cardiac effects of TZDs in non-T2D populations. Horio et al. investigated 30 non-diabetic patients with essential hypertension and found no change in absolute values of LA and LVDD after six-month treatment with pioglitazone [10]. Our findings contradict this study, which may be because of a completely different study population.

One patient in the pioglitazone group developed a significant mitral regurgitation during the treatment, but this was proved to be a reversible condition. An echocardiography examination six months after discontinuation of the pioglitazone medication showed regression of the mitral regurgitation [3]. This finding is interesting, but of anecdotal character and should be viewed with caution. We have previously speculated about the explanation of this finding [3]. One plausible hypothesis is that the fluid retention caused left ventricular dilatation, which in turn resulted in mitral annular dilatation. The mitral annular dilatation then led to inadequate mitral leaflet coaptation.

In the last few years, two TZDs have been available on the market, namely pioglitazone and rosiglitazone. Recent meta-analyses point to differences in the effect of these two drugs on cardiovascular outcomes. There is evidence that the increased risk of developing congestive heart failure is greater with rosiglitazone [11]. The increased risk of developing heart failure with pioglitazone has not been associated with an increase in mortality [12,13]. Pioglitazone has also been shown to reduce incidence of ischemic vascular events, which is not true for rosiglitazone.

Echocardiography is considered "gold standard" method, in daily clinical practice, for evaluating cardiac function. While an echocardiography examination is broadly available and relatively inexpensive in the western World (150–250 Euros per examination in Sweden), the screening costs for larger groups of patients would be substantial. There is, therefore, a need for less expensive screening-tests. Taking our results into consideration, one could suggest that an initial assessment of a natriuretic peptide could be used for identifying individuals at risk of responding negatively to pioglitazone treatment. Serial monitoring of these peptides along with the clinical registration of weight and haemoglobin values during treatment could also be helpful to identify those few individuals, who should undergo an echocardiography evaluation and subsequently reduce the rate of unwanted side-effects.

## Conclusion

When choosing pioglitazone as an add-on treatment in patients with insufficiently controlled T2D, an increase in LVD vol by 11% and LA vol by 17% could be observed. This was parallel with an increase in natriuretic peptids. These changes in cardiac volume and natriuretic peptid-levels were not observed with insulin glargine. Larger randomised trials are warranted to confirm these findings. There is a need for tools to help clinicians to identify subsets of patients for whom this kind of therapy is likely to have particularly favourable/unfavourable effect, using readily identifiable clinical and laboratory factors. Knowledge is increasing in this field and hopefully the results will enable clinicians to select the proper therapy for individual patients in the future.

## Abbreviations

T2D: Type 2 diabetes; TZD: thiazolidinedione; BNP: brain natriuretic peptide; NT-proBNP: N-terminal pro brain natriuretic peptide; LVDD: left ventricular end-diastolic diameter; POST: posterior wall thickness; IVS: intraventricularseptum thickness; LVM: left ventricular mass; LVD vol: left ventricular end-diastolic volume; LVS vol: left ventricular end-systolic volume; LA vol: left atrial end-systolic volume; and EF: ejection fraction.

## Competing interests

Mozhgan Dorkhan has signed a speaker contract with Eli Lilly and received a scholarship from Sanofi-Aventis. Magnus Dencker has no conflict of interest to declare. Martin Stagmo has received speaker's fees from Sanofi-Aventis and through his spouse owns shares and stock options in NovoNordisk A/S.

## Authors' contributions

All authors participated in the design of the study. MDo and MDe wrote the manuscript. All authors edited and approved the final version of the manuscript.
